# Ratio of involved/uninvolved immunoglobulin quantification by Hevylite™ assay: clinical and prognostic impact in multiple myeloma

**DOI:** 10.1186/2162-3619-1-9

**Published:** 2012-04-23

**Authors:** Efstathios Koulieris, Panayiotis Panayiotidis, Stephen J Harding, Nikolitsa Kafasi, Dimitris Maltezas, Vassiliki Bartzis, Tatiana Tzenou, Maria Dimou, George Georgiou, Ladan Mirbahai, Arthur R Bradwell, Marie-Christine Kyrtsonis

**Affiliations:** 1 Haematology Section of 1st Department of Propedeutic Internal Medicine, Athens Medical School, Athens, Greece; 2 The Binding Site Group Ltd, Birmingham, UK; 3 Department of Immunology and Histocompatibility, Laikon University Hospital, Athens, Greece; 4 Department of Immunity and Infection, Medical School, University of Birmingham, Birmingham, UK

**Keywords:** Multiple myeloma, Prognosis, Serum immunoglobulin, Heavy/light chain measurements, HLCR

## Abstract

**Background:**

HevyLite™ is a new, recently developed method that facilitates separate quantification of the kappa- and lambda-bounded amounts of a given immunoglobulin (Ig). Using this method, we measured intact immunoglobulin (heavy/light chain; HLC) IgG-kappa, IgG-lambda, IgA-kappa, IgA-lambda individually, as well as their deriving ratios (HLCR) in a series of IgG or IgA multiple myeloma (MM) patients, to investigate and assess the contribution of these tests to disease evaluation.

**Patients and methods:**

HevyLite™ assays were used in sera from 130 healthy individuals (HI) and 103 MM patients, at time of diagnosis. In patients, the level of paraprotein was IgG in 78 (52 IgG-kappa, 26 IgG-lambda) and IgΑ in 25 (13 IgΑ-kappa, 12 IgΑ-lambda). Durie-Salmon and International Staging System stages were evenly distributed. Symptomatic patients (n = 77) received treatment while asymptomatic ones (n = 26) were followed. Patients' median follow-up was at 32.6 months. HLCR was calculated with the involved Ig (either G or A) as numerator.

**Results:**

In HI, median IgG-kappa was 6.85, IgG-lambda 3.81, IgA-kappa 1.19 and IgA-lambda 0.98 g/L. The corresponding median involving HLC values in MM patients were 25.8, 23.45, 28.9 and 36.4 g/L. HLC-IgG related to anemia, high serum free light chain ratio and extensive bone marrow infiltration, while high HLCR correlated with the same plus increased β2-microglobulin. In addition, increased HLCR and the presence of immunoparesis correlated with time to treatment. Patients with high HLCR had a significantly shorter survival (*p *= 0.022); HLCR retained its prognostic value in multivariate analysis.

**Conclusions:**

HLC and HLCR quantify the precise amount of the involved immunoglobulin more accurately than other methods; moreover, they carry prognostic information regarding survival in MM patients.

## Introduction

Multiple myeloma (MM) is characterized by bone marrow (BM) plasma cell infiltration and the presence of serum/urine monoclonal immunoglobulin (Ig). Disease manifestations and behavior vary widely, while survival ranges from a few months to more than a decade.

Serum monoclonal Ig quantification is part of MM diagnostic criteria, and one of Durie and Salmon (DS) staging system's risk factors [1]. It is also used for monitoring response and relapse. However, MM aggressiveness is not related to the amount of Ig secretion, and newer staging systems did not retain Ig quantity as a risk parameter [2].

The poor utility of IgG and IgA measurements may be related to the following:1) Changes in hematocrit and blood volume occur in MM leading serum Ig concentrations to vary by 50% or more, independently of changes in tumor production [3]; 2) Serum IgG concentrations are affected by variable clearance rates, so measurements do not reliably relate to tumor production. For example, IgG is recycled many times by neonatal Fc receptors (FcRn), therefore at normal serum concentration, its half-life is extended from 3 to 21 days. At higher IgG concentration, FcRn receptors are saturated, so IgG half-life is reduced [4]. Thus, clearance rates vary with different IgG levels. Similar arguments may apply to IgA measurements [5]. When measuring IgA, proteins such as transferrin can co-migrate in protein electrophoresis gels, contributing to falsely elevated results [6].

A new method was recently developed and validated for the separate quantification of the kappa- and lambda-bounded amounts of circulating IgG and IgA [7,8]. This was achieved by developing antibodies targeting unique junctional epitopes between the heavy and light chains of each Ig molecule. Immunoassay such as Hevylite™ using these antibodies as target has been manufactured for the analysis of Ig heavy/light chain (HLC) pairs. The aforementioned assay allows the quantification of the absolute value of the involved IgGκ, IgGλ, IgAκ and IgAλ separately, along with their deriving ratios (HLCR): IgGκ/IgGλ, IgGλ/IgGκ, IgAκ/IgAλ and IgAλ/IgAκ [7].

In paraproteinemias, the "classical" nephelometric quantification of total "intact" Ig comprises the paraprotein *per se*, and the remaining kappa- and lambda-bounded Ig of the same class. With the Hevylite™, if the involved Ig (iHLC) is calculated as a nominator, the ratio's (HLCR) denominator represents only polyclonal intact Ig of the same class (p-HLC), bounded to the "non-restricted" light chain; the nominator (iHLC) is made up of the monoclonal protein and the remaining polyclonal Ig of the same class and light chain restriction, the value of which is much closer to the exact paraprotein amount than otherwise. In addition, increased polyclonal suppression leads to HLCR elevation because of the denominator's reduction. The purpose of this study was to investigate the contribution of HLCs and their deriving ratios (HLCR) in a series of MM patients at diagnosis.

## Patients and methods

### A. Patients

103 patients characterized by the presence of heavy chain paraprotein and referred to herein as "intact" Ig MM patients, were studied at diagnosis. There were 46 women and 57 men with a median age of 67. Of these, 31%, 27% and 42% were in DS stage Ι, ΙΙ and ΙΙΙ respectively, and 36%, 30%, and 34% were stages 1, 2 and 3 by ΙSS [9]. Anemia (Hb < 10 g/L) was present in 25% of all patients, with renal failure (creatinine ≥ 2 mg/dl) in 11%, thrombocytopenia (PLT < 140 × 10 [9]/L) in 13.5%, hypoalbuminaemia (alb < 3.5 g/L) in 20.8%, hypercalcemia in 12.6% and increased LDH (above normal) in 7%. 26% of these patients presented severe bone disease (osteolyses with fractures) and 48% had an extensive BM plasma cell infiltration (≥ 50%). Paraprotein type was determined by serum immunofixation, which was IgG in 78 patients (52 IgGκ, 26 IgGλ) and IgΑ in 25 (13 IgΑκ, 12 IgΑλ). Median total paraprotein level as routinely determined by nephelometry, which we called "total" paraprotein, was 31.78 g/L for IgG and 29.10 g/L for IgA at presentation. Median free light chain ratio (FLCR) was 9.44 in kappa-restricted patients and 19.62 in those who were lambda-restricted.

77 patients were already symptomatic or became symptomatic during follow up. Symptomatic patients were treated with conventional modalities. The 26 asymptomatic patients were only followed. Patients' median follow-up was for 32.6 months.

### B. Methods

HLC were determined nephelometry on a Dade Behring BN II Nephelometer at the Binding Site laboratory in Birmingham, U.K. Polyclonal sheep antibodies (Hevylite™, Binding Site, U.K.) were used as described [6]. The HLCRs were calculated with the involved intact Ig as numerator, and the polyclonal intact Ig of the same class as denominator (IgGκ/p-IgGλ, IgGλ/p-IgGκ, IgAκ/p-IgAλ and IgAλ/p-IgAκ). Measurements were reproducible.

Frozen sera from the aforementioned 103 patients, drawn at the time of diagnosis, were retrospectively analyzed. In addition, HLC measurements performed in 130 healthy individuals (HI) were used for control values.

We called the presence of hypogammaglobulinemia of uninvolved Igs systemic immunoparesis (SI); it was defined using our laboratory lower cut-off values as IgG < 6.9 g/L and/or IgM < 0.34 g/L in IgA-patients and IgA < 0.88 g/L and/or IgM < 0.34 g/L in IgG-patients.

Immunoparesis of the same class (ISC) was defined as any p-HLC value below the 95th percentile range of p-HLCs in HI sera. Thus, IgGλ, IgGκ, IgAλ and IgAκ patients were considered to have ISC when their p-IgGκ, p-IgGλ, p-IgAκ and p-IgAλ were below 4.03 g/L, 1.97 g/L, 0.48 g/L and 0.38 g/L respectively.

"High" HLCR was defined as any value above median.

HLCs and HLCR values were compared with disease parameters such as beta2-microglobulin (β2Μ), haemoglobin (Hb), serum albumin (alb), creatinine (cr), hypercalcemia, LDH, severe bone disease, serum free light chains (sFLC) and their ratio (sFLCR), bone marrow (BM) infiltration and survival.

Statistical analysis was performed using SPSS v15.0. Hazard ratios and the prognostic significance of "high" HLCR were determined by univariate Cox regression analysis. Kaplan Meier method was used for pictorial representation of survival and time to treatment. Categorical variables were compared with the chi square test (x^2^).

All sera were obtained with informed consent and the use of frozen sera for the retrospective evaluation of HLCs was approved by the local ethical committee.

## Results

### Hevylite™ assays in HI

In 130 HI, IgG-kappa ranged from 4.03 to 9.78 g/L (median 6.85), IgG-lambda from 1.97 to 5.71 (median 3.81), IgA-kappa from 0.48 to 2.82 (median 1.19) and IgA-lambda from 0.36 to 1.98 (median 0.98). The ratio IgG-kappa/IgG-lambda ranged from 0.98 to 2.75 (median 1.87), the IgG-lambda/IgG-kappa from 0.37 to 0.893 (median 0.54), the IgA-kappa/IgA-lambda from 0.8 to 2.04 (median 1.27) and the IgA-lambda/IgA-kappa from 0.49 to 1.256 (median 0.79) (Table 1).

**Table 1:** HLC-IgG, -IgA and HLCR Results in 130 Healthy Individuals

	Median	95th Percentile range
IgG kappa (g/L)	6.85	4.03-9.78
IgG lambda (g/L)	3.81	1.97-5.71
IgG kappa/IgG lambda HLCR	1.87	0.98-2.75
IgG lambda/IgG kappa HLCR	0.54	0.37-0.893
IgA kappa (g/L)	1.19	0.48-2.82
IgA lambda (g/L)	0.98	0.36-1.98
IgA kappa/IgA lambda HLCR	1.27	0.80-2.04
IgA lambda/IgA kappa HLCR	0.79	0.49-1.256

### Hevylite™ assays in multiple myeloma patients at diagnosis

Median iHLC in IgG patients was 25.61 g/L; it ranged from 1.80 to 101 g/L (median 25.8) for iIgG-kappa, and from 2.27 to 86.3 g/L (median 23.45) for iIgG-lambda. Median iHLC in IgA patients was 34.9 g/L, ranging from 5.59 to 71.6 g/L (median 28.9) in iIgA-kappa and from 7.23 to 60.4 g/L (median 36.4) in iIgA-lambda patients, respectively. Corresponding correlations with total IgG and IgA were r = 0.8 and 0.9. All patients had abnormal HLCR at diagnosis. The median HLCR in IgG-MM was 21.47, and 72.42 in IgA-MM.

### Correlations between disease variables and HLC measurements

Involved HLC-IgG absolute values above median correlated with Hb ≤ 10 g/L (*p *= 0.019), BM infiltration > 50% (*p *= 0.011) and sFLCR above median (*p *= 0.017), while iHLC- IgA did not correlate to any disease parameters. "High" HLCR correlated with β2Μ > 3.5 mg/L (*p *= 0.024), Hb ≤ 10 g/L (*p *= 0.003), BM infiltration > 50% (*p *= 0.002) and sFLCR above median (*p *= 0.004). SI was present in 76 out of 103 patients, ISC in 83/103 and both in 63/103.

### Relationship between HLC measurements and time to progression

**Figure 1:**
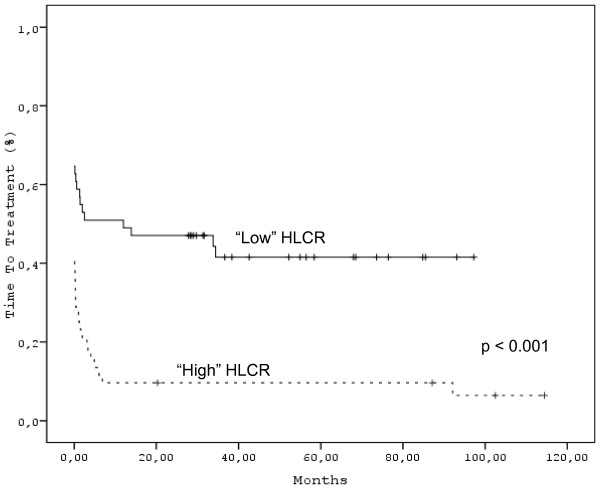
**"High" or "Low" HLCR in MM patients at diagnosis**.

**Figure 2:**
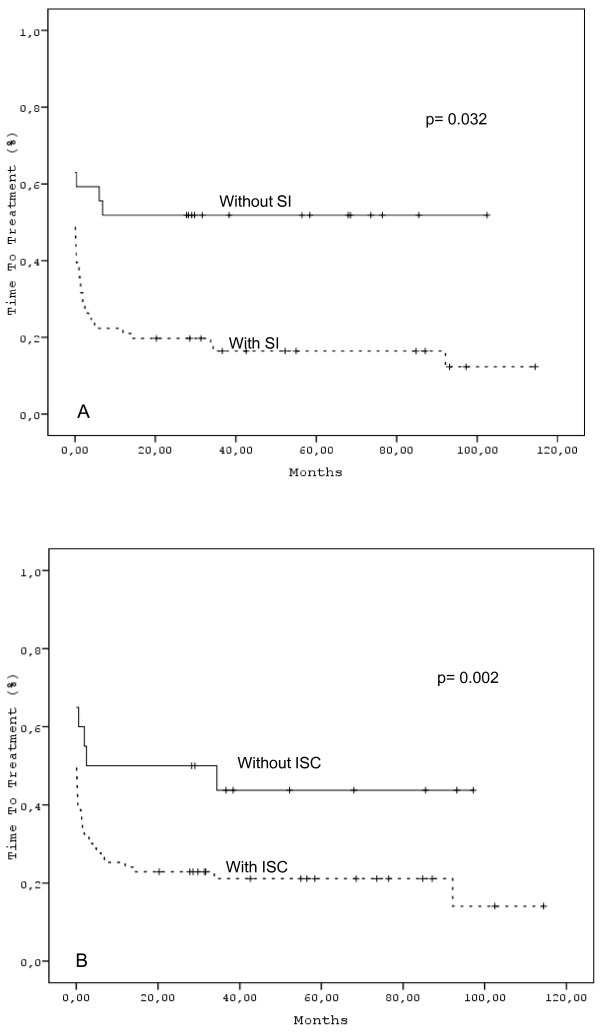
**Time to treatment according (A) to the presence or absence of ISC and (B) SI**.

As expected, "high" HLCR correlated with time to treatment (*p *< 0.001) (Figure 1). This was indeed also the case for "total" paraprotein. ISC was also associated with shorter time to treatment (*p *= 0.032). The same was true for SI (*p *= 0.002).(Figure 2A &2B).

### Relationship between HLC measurements and overall survival

**Figure 3:**
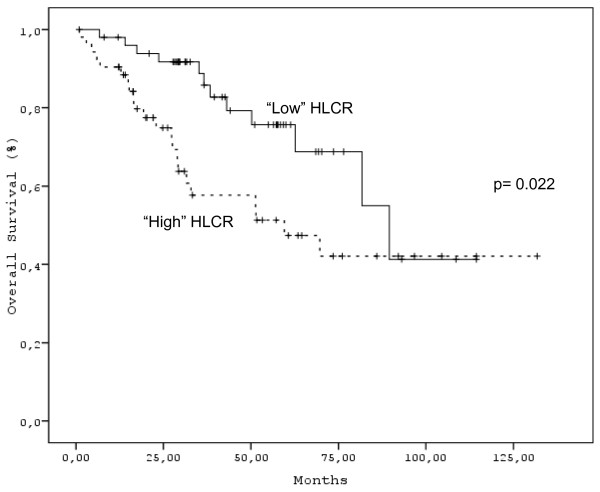
**Overall survival in patients with "High" or "Low" HLCR**.

Disease variables with an adverse prognostic impact on survival were Durie and Salmon stage (*p *= 0.046), ISS stage (*p *< 0.001), β2Μ above 3.5 mg/L (*p *< 0.001), Hb ≤ 10 g/L (*p *= 0.004), platelet counts ≤ 140 × 10 [9]/L (*p *< 0.001), alb < 3.5 g/L (*p *= 0.015), Cr > 2 mg/dL (*p *< 0.001), BM plasma cell infiltration > 50% (*p *< 0.001), sFLCR above median (*p *= 0.022) and "high" HLCR values (*p *= 0.022) (Figure 3). On the contrary, IgG and IgA paraprotein levels, HLC, SI, ISC and IgM levels, were not of any significance with regard to prognosis. In multivariate analysis, only platelet count, β2M and HLCR remained significant.

## Discussion

In the present study the clinical utility of the new intact immunoglobulin heavy chain/light chain (Hevylite™) method was assessed in a series of "intact" Ig MM patients, studied from the time of diagnosis. Sex, age, stage groups and clinical manifestations were representative and equivalent to any other non-selected MM patients cohort, with the exclusion of light chain and non-secretory patients. Paraprotein quantification with Hevylite™ assays correlated with "total" Ig measurements, as expected; in addition, it revealed the exact amount of remaining polyclonal Ig of paraprotein class, as well as the patient's light chain restriction.

We showed here that iHLC-IgG related to parameters of disease activity, severity and tumor burden, including anemia, sFLCR and extensive bone marrow infiltration, while HLCR with the same plus increased β_2_Μ. Usually, when such correlations are obtained with the aforementioned markers, relationship with prognosis is also found. In our study, this was proven true, and the ratio of involved/uninvolved Ig (HLCR) correlated with time to treatment, and most importantly with outcome; thus, patients with "high" HLCR had a significantly shorter survival. The improved prognostic information given by HLCR compared to "total" Igs probably relates to biased classical Igs measurement in some cases, as discussed earlier; also because polyclonal, non-tumour Ig are included in "total" Ig quantification. HLCR importance for progression free survival was recently reported by others [10]. We additionally confirmed that polyclonal immunosuppression (in the form of systemic immunoparesis or immunoparesis of the same class or both) was related to time to treatment [11]; although it had no impact on overall survival. The importance of IgM with regard to survival was recently reported by Perosa *et al *[12]; nevertheless, IgM levels determined by conventional method did not carry any prognostic information in this series.

Myeloma cells from individual bone marrow biopsies can produce either "intact" immunoglobulins, free light chains, or both [13], allowing presumption that the prognostic information provided by the new M-component-based markers (FLC and HLC) could be complementary; however, in this study, sFLCR and HLCR strongly correlated and their respective prognostic power was equivalent.

Both albumin and beta_2_-microglobulin (β_2_Μ) are linked to IgG metabolism via FcRn molecules. β_2_Μ is the light chain component of the heterodimeric FcRn receptor, while albumin is recycled via the same molecule [7]. We speculate that some of the clinical utility of albumin and β_2_Μ in the ISS may be linked to IgG metabolism. In our study, HL IgG was related to albumin but not to β_2_Μ, although "high" HLCR correlated with increased β_2_Μ.

Other disease parameters with an adverse prognostic impact in this series were, as expected [2], ISS stage [9], FLCR above median [14], β_2_Μ above 5, 5 mg/L [15], albumin below 3.5 g/L [16], haemoglobin below 10 g/L, platelet counts below 140 × 10 [9]/L [17], serum creatinine above 2 mg/L [18] and extensive BM plasma cell infiltration. Significantly, in multivariate analysis only platelets, β2M and HLCR retained their statistical significance.

In conclusion, we provide evidence that HLCR values correlate with adverse markers and are themselves independently prognostic in patients with MM. Thus, by having the quadruple advantage of determining separately the kappa- and lambda-bounded amounts of circulating IgG and IgA, of assessing the light chain restriction as well as the depth of suppression of the polyclonal Ig of the same and of the uninvolved class, and of contributing to prognostication, the new intact Ig HLC assay, Hevylite™, may conceivably replace "classical" total Ig quantitative measurements in the future. Retrospective and prospective analyses of larger patient cohorts are necessary for further validation.

## Competing interests

A.R.B. is a director and shareholder of The Binding Site Ltd. S.J.H. and L.M. are employees of Binding Site. EK, DM, NK, VB, TT, MD, GG, PP and MCK, declare that they have no competing interests.

## Authors' contributions

Contribution: EK provided the clinical samples, reviewed patients' files and wrote the paper. M-CK followed patients, wrote the manuscript, provided the clinical samples, and supervised the study; SJH analysed the data and wrote the manuscript. DM analysed the data and co-authored the manuscript. NK supervised total Igs and FLC measurements in Greece and co-authored the manuscript. VB, TT, MD and GG followed patients, PP followed patients, provided facilities for sample storage and co-authored the paper, LM analysed the data, ARB wrote the manuscript and developed the study hypothesis. All authors read and approved the final manuscript.
